# Endoscopic Endonasal Odontoidectomy and Occipitoaxial Fixation for Basilar Invagination With Platybasia and Atlas Occipitalization Associated With Trichorhinophalangeal Syndrome

**DOI:** 10.7759/cureus.116576

**Published:** 2026-09-20

**Authors:** Victor Correa-Correa, Edgar F Higuera-Gonzalez, Kevin S Toache, Diego Molina-Botello, Christian Ruiz-Flores, Eduardo Ichikawa-Escamilla

**Affiliations:** 1 Neurosurgery, Hospital de Especialidades, Centro Médico Nacional Siglo XXI, Instituto Mexicano del Seguro Social, Mexico City, MEX

**Keywords:** basilar invagination, osteochondrodysplasias, platybasia, skull base, spinal cord disease

## Abstract

Basilar invagination is a craniovertebral junction deformity characterized by the upward migration of the odontoid process toward the foramen magnum. This condition is frequently associated with congenital anomalies such as platybasia and atlas occipitalization. On the other hand, trichorhinophalangeal syndrome is a rare autosomal dominant skeletal dysplasia characterized by distinctive craniofacial features, brachydactyly, and abnormalities in endochondral ossification.

This study describes the surgical treatment performed in a 39-year-old man with genetically confirmed trichorhinophalangeal syndrome and basilar invagination with platybasia and atlas occipitalization. These malformations caused severe ventral cervicomedullary compression. Computed tomography angiography also revealed an aberrant vertebral artery with atlas occipitalization.

The procedure was performed in a single session, combining endonasal endoscopic odontoidectomy for immediate anterior decompression with posterior occipitoaxial fixation (C0-C2). To reduce the risk of vascular injury due to the vertebral artery anomaly, C2 translaminar screws were used instead of pedicle or transarticular screws. This approach achieved complete decompression, short stabilization, and rapid, sustained neurological recovery, highlighting hybrid minimally invasive strategies for complex craniovertebral junction pathology.

Key aspects are the following: Firstly, endoscopic endonasal odontoidectomy is the standard for ventral decompression, providing a direct route, less oropharyngeal morbidity, and faster recovery. Secondly, in instability, C0-C2 occipitocervical fixation is the minimal sufficient posterior stabilization, avoiding unnecessary multilevel fixation and preserving subaxial motion. Lastly, translaminar instrumentation is advised to reduce vascular injury risk in atypical vertebral artery anatomy, common in craniovertebral junction malformations.

## Introduction

Basilar invagination is a complex craniovertebral junction deformity characterized by the superior migration of the odontoid process toward the foramen magnum, leading to ventral compression of the brainstem and upper cervical spinal cord [1,2]. This condition is frequently associated with atlantoaxial instability and may coexist with congenital anomalies such as platybasia and atlas occipitalization [1-3]. Patients with congenital craniovertebral junction anomalies exhibit a significantly higher prevalence of vertebral artery variations than the general population, making preoperative vascular assessment essential prior to instrumentation. Three-dimensional computed tomography (CT) angiography has revealed a high incidence of anomalous V3 segment anatomy in patients with basilar invagination, which substantially increases the risk of vertebral artery injury during posterior fixation [4,5].

Trichorhinophalangeal syndrome is a rare autosomal dominant skeletal dysplasia caused by pathogenic variants in the TRPS1 gene and characterized by distinctive craniofacial features, brachydactyly, and abnormalities of endochondral ossification [6,7]. Although basilar invagination is not typically associated with trichorhinophalangeal syndrome, impaired skeletal development involving the skull base may predispose affected patients to craniovertebral junction instability. Only isolated cases describing this association have been reported, and the optimal surgical management remains undefined [6-8]. The surgical treatment of irreducible ventral cervicomedullary compression has evolved significantly over the past two decades. Endoscopic endonasal odontoidectomy has emerged as a minimally invasive alternative to the traditional transoral approach, offering comparable neurological outcomes while reducing approach-related morbidity [9-13]. Posterior stabilization remains the cornerstone of treatment for craniovertebral instability, and when vertebral artery anatomy precludes the safe placement of pedicle or transarticular screws, C2 translaminar fixation provides a safe and biomechanically reliable alternative [3,8,14].

In this study, we present the surgical treatment of a patient with trichorhinophalangeal syndrome and multiple associated malformations. Surgery at the craniocervical junction is complex, as it requires extensive knowledge of the spine and the skull base; therefore, the case presented here is even more complex and illustrates the importance of an individualized anatomical evaluation that can guide the appropriate selection of the safest decompression and stabilization strategy.

## Case presentation

A 39-year-old man with genetically confirmed trichorhinophalangeal syndrome and no significant medical history presented with a three-month history of neck and low back pain, paresthesias in all four extremities, gait instability, and bilateral foot numbness while walking. Despite treatment with nonsteroidal anti-inflammatory drugs, his symptoms progressed and increasingly limited his daily activities. Neurological examination revealed restricted cervical range of motion, generalized hyperreflexia (4+) in both the upper and lower extremities, bilateral ankle clonus, and preserved muscle strength (Medical Research Council [15] grade 5/5). Sensory examination was normal, with no bowel or bladder dysfunction. He remained independently ambulatory. His modified Japanese Orthopaedic Association (mJOA) score was 13 (an 18-point scale assessing upper extremity motor function, sensory function, and sphincter control in cervical myelopathy, with lower scores indicating greater disability) [16], and his Nurick grade was 3 (a 0-5 scale grading gait disability attributable to cervical myelopathy, with higher grades indicating greater disability) [17].

Radiological assessment included CT, magnetic resonance imaging (MRI), and CT angiography. CT demonstrated basilar invagination with platybasia and complete atlas occipitalization, resulting in severe narrowing of the foramen magnum (anteroposterior diameter, 28 mm; transverse diameter, 25 mm), compared with normal adult reference values of approximately 32-36 mm anteroposteriorly and 27-31 mm transversely. MRI revealed severe ventral compression of the cervicomedullary junction with associated intramedullary T2 hyperintensity, consistent with chronic compressive cervical myelopathy (Figure 1). CT angiography identified an anomalous course of the V3 segment of the vertebral artery related to atlas assimilation, which was carefully considered during surgical planning.

**Figure 1:**
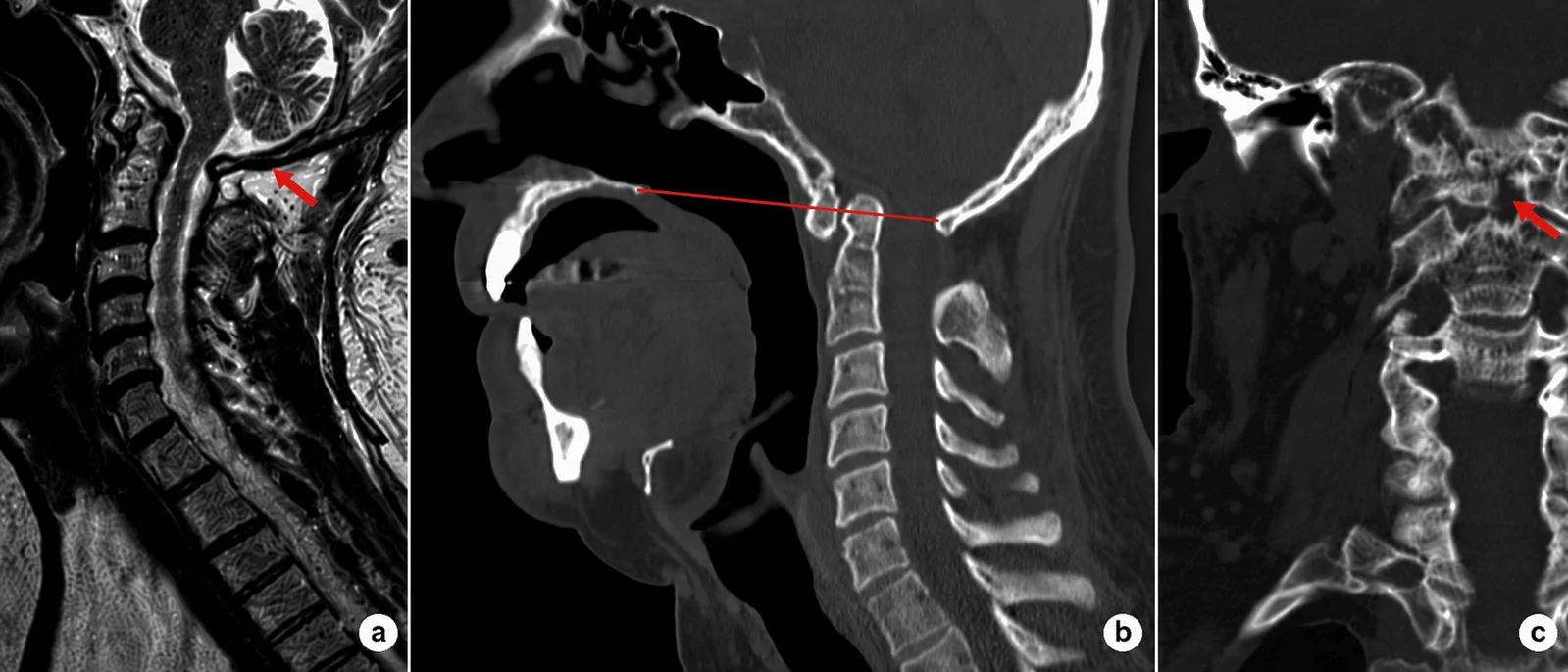
Preoperative radiological assessment of basilar invagination with platybasia and occipitalization of the atlas Preoperative MRI (a) and CT images (b, c) demonstrating basilar invagination associated with platybasia and atlas occipitalization. (a) Sagittal T2-weighted MRI showing superior migration of the odontoid process with severe ventral compression of the medulla and upper cervical spinal cord; the red arrow indicates the point of maximal compression at the cervicomedullary junction. (b) Sagittal CT reconstruction confirming ascent of the odontoid process above Chamberlain's line and narrowing of the upper cervical spinal canal; the red line indicates the odontoid process displaced superiorly above Chamberlain's line. (c) Coronal CT reconstruction demonstrating occipitoatlantal fusion and altered craniocervical alignment; the red arrow indicates atlas occipitalization. MRI: magnetic resonance imaging; CT: computed tomography

Given the patient's myelopathy, irreducible ventral cervicomedullary compression, and craniovertebral junction anatomy, a single-stage circumferential approach was planned, consisting of endoscopic endonasal odontoidectomy for ventral decompression followed by posterior decompression and occipitoaxial stabilization. The anterior stage was performed with the patient in the supine position under general anesthesia using an endoscopic endonasal approach with electromagnetic neuronavigation. After exposure of the clivus and the assimilated anterior arch of C1, sequential drilling was performed to remove the clivus, anterior arch of C1, and odontoid process until adequate ventral decompression was achieved, as confirmed by visualization of a pulsatile dural sac. Reconstruction was performed using a musculomucosal flap, hemostatic material, and fibrin sealant (Figure 2). During the same anesthetic session, the patient was repositioned prone for C1 laminectomy, limited foramen magnum decompression, and occipitoaxial fixation using an occipital plate and bilateral C2 translaminar screws. C1 instrumentation was avoided because of complete atlas occipitalization and the anomalous vertebral artery anatomy (Figure 3). Posterolateral fusion was performed using a bone graft substitute. The total operative time was 280 minutes, with an estimated blood loss of 200 mL.

**Figure 2:**
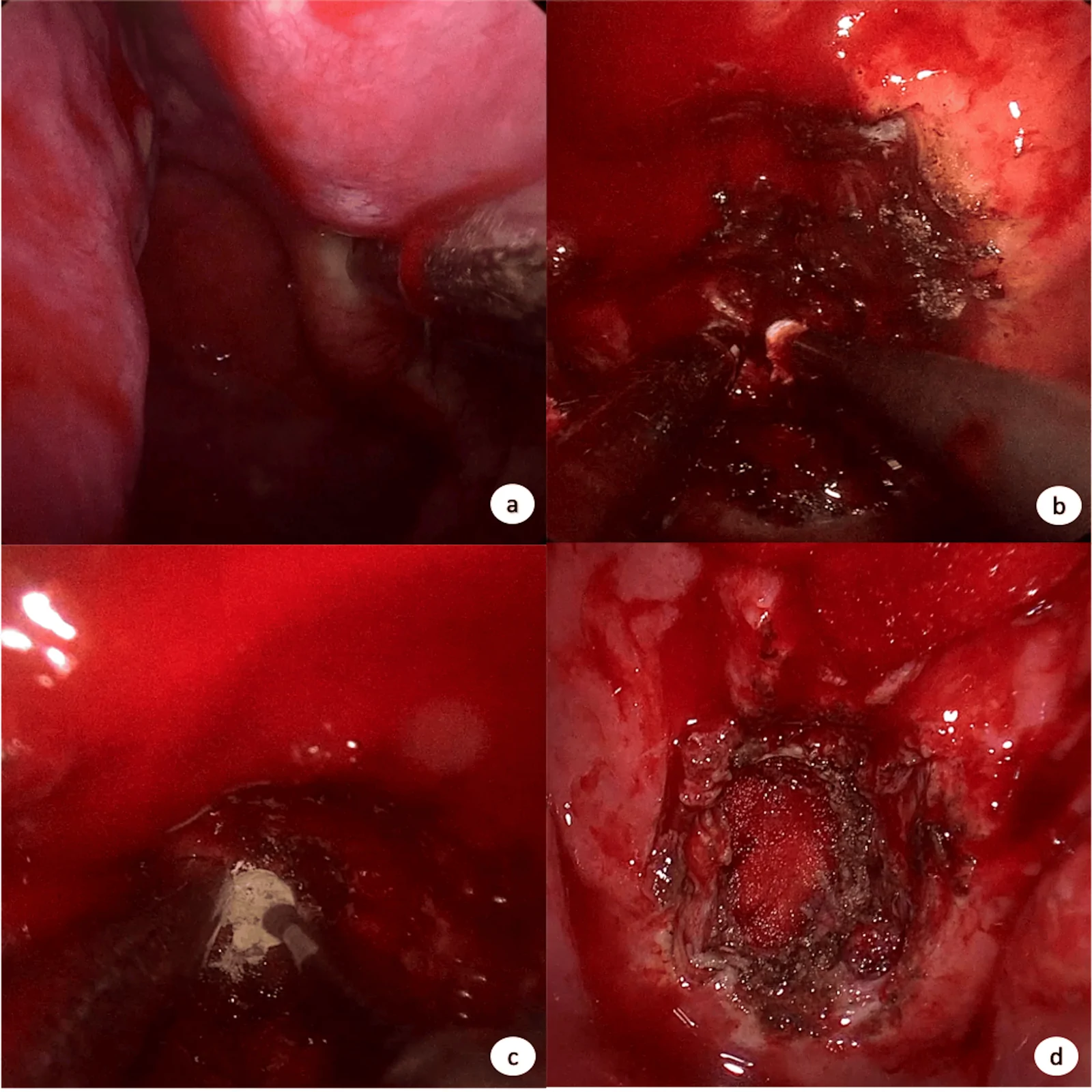
Intraoperative endoscopic views of the endoscopic endonasal odontoidectomy (a) Endoscopic view during the nasal phase of the endoscopic endonasal approach for odontoidectomy, demonstrating establishment of the surgical corridor toward the craniovertebral junction. (b) Endoscopic resection of the anterior arch of C1 to expose the odontoid process. (c) Progressive drilling of the odontoid process using a high-speed drill to achieve ventral decompression. (d) Final endoscopic view after the completion of the odontoidectomy, demonstrating hemostasis and closure of the surgical corridor.

**Figure 3:**
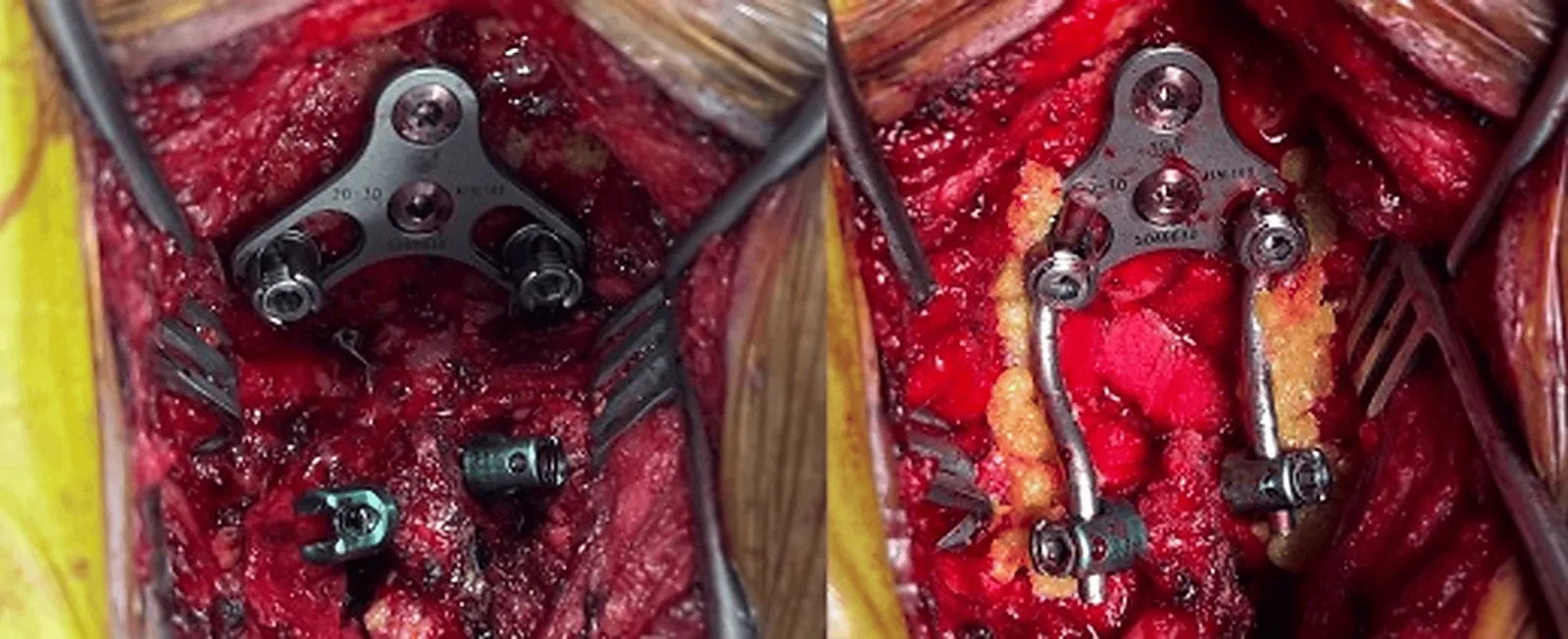
Posterior occipitoaxial fixation The patient is positioned prone with the head neutral in the coronal plane and 5° ventral flexion in the sagittal plane, confirmed by fluoroscopy, with an occipitovertebral angle of 135° to optimize sagittal vertical balance. Through a suboccipital and posterior cervical approach, occipitoaxial fixation is performed using rods and screws (C2 translaminar screws of 20 and 24 mm × 3.5 mm and an occipital plate with two 3.5 × 8 mm keel screws), with posterolateral bone graft over decorticated occipitovertebral bone.

The postoperative course was uneventful. The patient was extubated immediately, resumed oral intake on the day of surgery, and remained neurologically intact, with no cerebrospinal fluid leakage, wound complications, dysphagia, or cranial nerve deficits. Postoperative CT and MRI confirmed complete odontoidectomy, adequate ventral cervicomedullary decompression, and appropriate positioning of the occipitoaxial instrumentation (Figure 4). Small bone fragments adjacent to the dura were intentionally left in situ because they moved with cerebrospinal fluid pulsations and their removal was considered to carry a risk of dural injury and cerebrospinal fluid leakage. The patient was discharged on postoperative day 2. Cervical pain and gait instability improved, while the neurological examination remained stable, with an mJOA score of 17 and a Nurick grade of 1. At the four-month follow-up, the clinical improvement persisted, gait impairment had resolved, and there was no instrumentation failure or radiographic instability. Cervical range of motion was reduced in flexion-extension, with this limitation remaining consistent across the intact subaxial segments.

**Figure 4:**
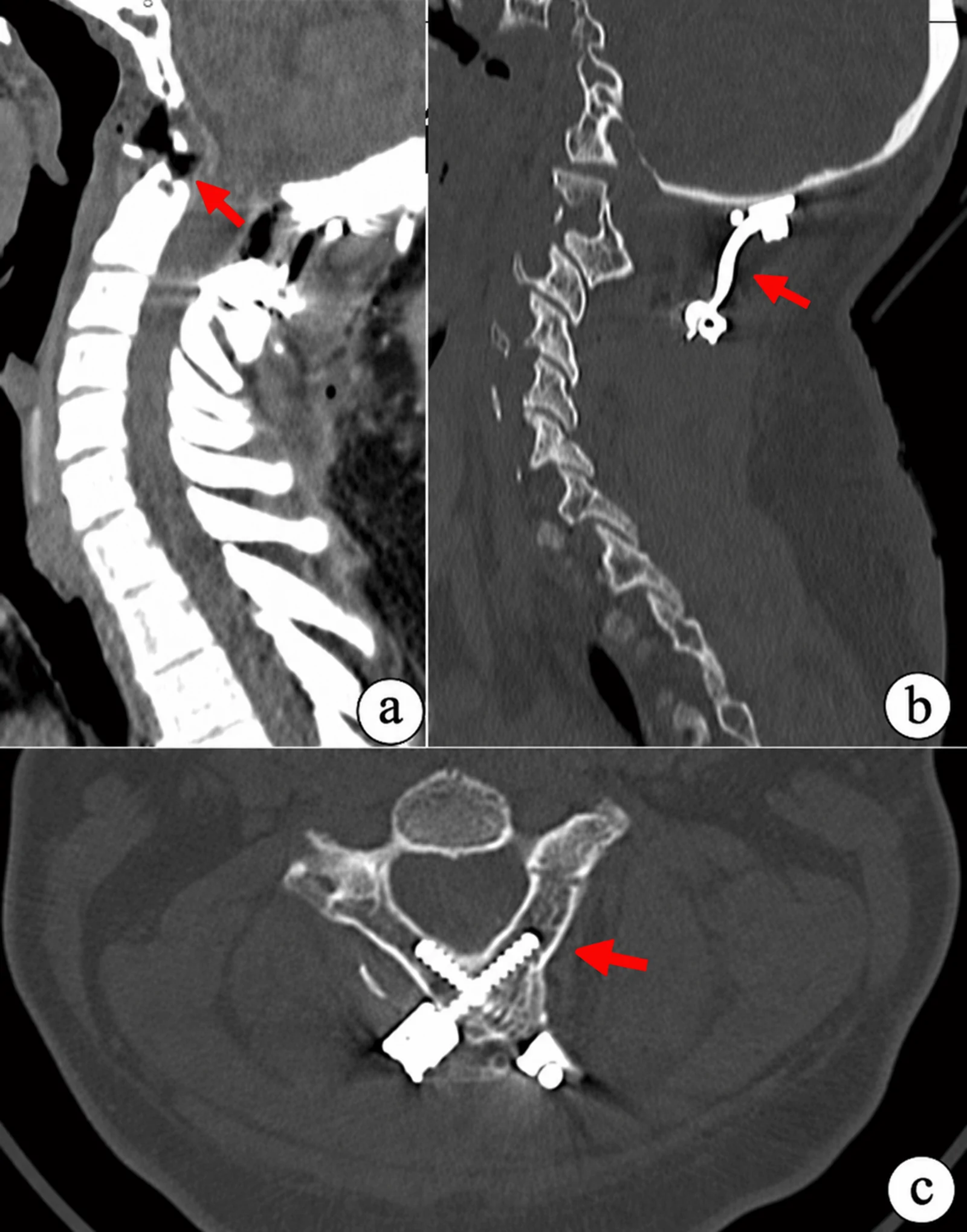
Postoperative CT images demonstrating complete ventral decompression and adequate occipitocervical stabilization Postoperative CT images demonstrating adequate decompression and stable posterior fixation. (a) Sagittal image showing complete odontoid resection and restoration of the ventral subarachnoid space at the cervicomedullary junction; the red arrow indicates the tip of the odontoidectomy defect. (b) Sagittal bone-window reconstruction demonstrating occipitoaxial fixation with an occipital plate and C2 translaminar screw construct, maintaining anatomic reduction; the red arrow indicates the fixation rod. (c) Axial CT image at the C2 level confirming appropriate position, trajectory, and symmetry of the translaminar screws without evidence of canal violation; the red arrow indicates the right C2 translaminar screw. CT: computed tomography

## Discussion

Basilar invagination associated with genetically confirmed trichorhinophalangeal syndrome is rare, and the optimal surgical management remains poorly defined [6]. Although trichorhinophalangeal syndrome is a skeletal dysplasia caused by abnormal endochondral ossification, craniovertebral junction involvement has rarely been reported [7]. Consequently, the evidence guiding surgical decision-making is limited, particularly in patients with atlas occipitalization, platybasia, and vertebral artery anomalies [18]. In this case, preoperative evaluation allowed for a circumferential strategy that achieved ventral decompression, stabilization, and neurological recovery, underscoring the importance of individualized surgical planning for syndromic craniovertebral junction deformities rather than standardized treatment algorithms.

Surgical treatment of basilar invagination has evolved over two decades. While transoral odontoidectomy was the standard approach for irreducible ventral cervicomedullary compression, advances in endoscopic skull base surgery have shifted the paradigm toward the endoscopic endonasal approach in selected patients [9,10]. Compared with the transoral approach, the endoscopic endonasal approach provides a superior trajectory to the odontoid process and panoramic visualization and avoids tongue retraction and palate splitting, thereby reducing morbidity [10,11]. Systematic reviews and contemporary series have shown similar neurological outcomes, with earlier extubation, faster oral intake, shorter hospitalization, and fewer airway-related complications [12]. Direct comparative data support these observations: in a case series contrasting endoscopic endonasal with transoral odontoidectomy for non-neoplastic craniovertebral junction disease, the endonasal approach required a longer operative time (238 vs. 141 minutes; p<0.02) but was associated with fewer complications (no complications in the endonasal group versus dysphonia, dysphagia, and one cerebrospinal fluid leak in the transoral group) along with earlier extubation, faster return to oral intake, and shorter hospital stay, with comparable neurological outcomes between approaches [9]. Although technically more demanding and associated with a steeper learning curve, the endoscopic endonasal approach reduces soft tissue morbidity, consistent with our patient's uncomplicated recovery and same-day oral intake. In our patient, the location of the odontoid process and craniovertebral anatomy made the endoscopic endonasal approach advantageous, enabling complete decompression and an uncomplicated recovery [9].

Although posterior reduction and fixation alone are increasingly accepted for basilar invagination, anterior decompression remains essential in selected cases of irreducible ventral brainstem compression [2,3]. Treatment should be individualized based on deformity reducibility, ventral neural compression, and patient-specific anatomy. In this case, persistent ventral cervicomedullary compression with complete occipitalization of the atlas precluded indirect decompression alone, supporting a circumferential strategy. Posterior stabilization was individualized. Preoperative CT angiography demonstrated an anomalous V3 segment vertebral artery, making conventional C1 instrumentation hazardous. Accordingly, occipitoaxial fixation with bilateral C2 translaminar screws minimized the risk of vascular injury. Anatomical and clinical studies support translaminar fixation as an effective alternative when pedicle anatomy or vertebral artery variants limit conventional screw placement. 

The association between trichorhinophalangeal syndrome and craniovertebral pathology merits special attention. Pathogenic variants affecting the TRPS1 gene alter chondrocyte differentiation and endochondral ossification, leading to widespread skeletal abnormalities [6,7]. While the syndrome is classically characterized by distinctive craniofacial features, brachydactyly, and short stature, abnormalities affecting the skull base and upper cervical spine may represent an underrecognized manifestation of the same developmental process [7]. Experimental models have shown that disruption of TRPS1 signaling produces abnormal cartilage maturation and premature ossification, providing a plausible biological mechanism for the development of platybasia, atlas assimilation, and subsequent basilar invagination [6,7]. Our findings broaden the clinical spectrum of trichorhinophalangeal syndrome and suggest that patients presenting with cervical myelopathy symptoms should undergo careful evaluation of the craniovertebral junction, as early recognition may allow timely surgical intervention before irreversible neurological injury occurs [7,18]. Postoperatively, the patient demonstrated improved cervical alignment, with a more physiological head position and reduced forward flexion. However, the characteristic craniofacial features and widespread skeletal abnormalities associated with trichorhinophalangeal syndrome, including sparse scalp hair, a bulbous nasal tip, and brachydactyly, remained unchanged, as expected given their congenital and non-progressive nature.

Beyond its rarity, this case offers valuable lessons. Preoperative CT angiography is indispensable for defining vascular and bony anatomy prior to instrumentation. The endoscopic endonasal approach is a minimally invasive alternative for upper ventral compression. Customized fixation allows stabilization to be achieved while minimizing the risk of vertebral artery injury. Despite the short follow-up period, this case contributes clinical evidence; multicenter studies are needed to define trichorhinophalangeal syndrome-associated abnormalities and treatment algorithms.

## Conclusions

In this patient, a single-anesthetic endoscopic endonasal odontoidectomy followed by C0-C2 stabilization achieved adequate ventral decompression and early neurological improvement. Detailed preoperative assessment of craniovertebral reducibility, bony anatomy, and vertebral artery course was central to selecting a safe, individualized operative strategy in the setting of genetically confirmed trichorhinophalangeal syndrome.
